# Introgressive Hybridization of *Schistosoma haematobium* Group Species in Senegal: Species Barrier Break Down between Ruminant and Human Schistosomes

**DOI:** 10.1371/journal.pntd.0002110

**Published:** 2013-04-04

**Authors:** Bonnie L. Webster, Oumar T. Diaw, Mohmoudane M. Seye, Joanne P. Webster, David Rollinson

**Affiliations:** 1 Wolfson Wellcome Biomedical Laboratories, Department of Life Sciences, The Natural History Museum, Cromwell Road, London, United Kingdom; 2 Institut Sénégalais de Recherches Agricoles, ISRA route des Hydrocarbures, Bel Air, Dakar, Sénégal; 3 Department of Infectious Disease Epidemiology, Imperial College Faculty of Medicine (St Mary's Campus), Norfolk Place, London, United Kingdom; Centers for Disease Control and Prevention, United States of America

## Abstract

**Background:**

Schistosomes are dioecious parasitic flatworms, which live in the vasculature of their mammalian definitive hosts. They are the causative agent of schistosomiasis, a disease of considerable medical and veterinary importance in tropical and subtropical regions. Schistosomes undergo a sexual reproductive stage within their mammalian host enabling interactions between different species, which may result in hybridization if the species involved are phylogenetically close. In Senegal, three closely related species in the *Schistosoma haematobium* group are endemic: *S. haematobium*, which causes urogenital schistosomiasis in humans, and *S. bovis* and *S. curassoni*, which cause intestinal schistosomiasis in cows, sheep and goats.

**Methodology/Principal Findings:**

Large-scale multi-loci molecular analysis of parasite samples collected from children and domestic livestock across Senegal revealed that interactions and hybridization were taking place between all three species. Evidence of hybridization between *S. haematobium*/*S. curassoni* and *S. haematobium*/*S. bovis* was commonly found in children from across Senegal, with 88% of the children surveyed in areas of suspected species overlap excreting hybrid miracidia. No *S. haematobium* worms or hybrids thereof were found in ruminants, although *S. bovis* and *S. curassoni* hybrid worms were found in cows. Complementary experimental mixed species infections in laboratory rodents confirmed that males and females of each species readily pair and produce viable hybrid offspring.

**Conclusions/Significance:**

These data provide indisputable evidence for: the high occurrence of bidirectional hybridization between these *Schistosoma* species; the first conclusive evidence for the natural hybridisation between *S. haematobium* and *S. curassoni*; and demonstrate that the transmission of the different species and their hybrids appears focal. Hybridization between schistosomes has been known to influence the disease epidemiology and enhance phenotypic characteristics affecting transmission, morbidity and drug sensitivity. Therefore, understanding and monitoring such inter-species interactions will be essential for optimizing and evaluating control strategies across such potential hybrid zones.

## Author Summary

Schistosome blood flukes are transmitted through water contact and cause a severe debilitating disease in humans and their livestock. Understanding the biology and epidemiology of these parasites is essential to enable the development of better control strategies in endemic areas. Several species of schistosome exist and species barriers are normally maintained by differences in ecology, host specificity, and evolutionary history. However, hybridization between closely related species can occur if parasites infect the same definitive host. Here we report on the introgression between a human (*S. haematobium*) and two ruminant schistosomes (*S. bovis* and *S. curassoni*), the prevalence and distribution of their hybrids and the novel evidence for the presence of *S. haematobium*/*S. curassoni* hybrids in Senegalese children. Modern sampling and genotyping techniques have brought to light the extent of these hybridization events which could have been facilitated by the natural progression in farming, population (both human and livestock) movements and expansion, as well as changes in snail distribution, creating areas of sympatric transmission. Hybridization can lead to phenotypic characteristics that can influence disease epidemiology and control success, highlighting the importance of monitoring these evolving hybrid zones.

## Introduction

In recent years, developments in molecular tools and their use in epidemiological studies have revealed several cases of hybridization and introgression in plants and animals [1]–[8], although there are very few examples from metazoan parasites [7]–[8]. Hybridization can have a major impact on species diversification and adaptive radiation [9]–[12]. With regard to parasites and pathogens, this may have a crucial impact on disease epidemiology and evolution, affecting factors such as virulence, drug efficacy and response to control, and host range, potentially ultimately leading, in certain cases, to the evolution of new species of pathogens [13]–[14].

Schistosomiasis is a parasitic disease of considerable medical and veterinary importance throughout tropical and subtropical regions, caused by dioecious digeneans of the genus *Schistosoma*. Schistosomes infect more than 207 million people worldwide (the majority of whom are in sub-Saharan Africa) and cause chronic diseases that can lead to severe liver, intestinal and bladder pathology and death [15]. Schistosomiasis is also a disease affecting domestic livestock such as cattle, sheep and goats throughout Africa, the Middle East, Asia, and some countries bordering the Mediterranean Sea. Indeed it has been estimated that over 165 million cattle are infected worldwide with chronic infections resulting in hemorrhagic enteritis, anemia, emaciation and death [16].

Schistosomes have a two-host life cycle with an asexual stage occurring in an intermediate freshwater snail and a sexual stage within the definitive mammalian host from which eggs are voided in the urine or faeces of the infected individual, depending on the schistosome species involved. The sexual stage of these dioecious parasites enables interactions between male and female worms within their definitive hosts, while the asexual stage within the aquatic intermediate snail host gives rise to clonal larvae facilitating exposure and potential infection of any mammal in contact with the water.

Most *Schistosoma* species are host specific and geographically separated, which helps to maintain species barriers however, given the opportunity, heterospecific crosses between species can occur and have been demonstrated in controlled laboratory experiments [17]–[20]. Heterospecific crosses can lead to either parthenogenesis or hybridization depending on the phylogenetic distance of the species involved. Hybridization, with the production of generations of viable offspring, will result from heterospecific crosses between closely related species and viable hybrids have been observed experimentally to exhibit several enhanced phenotypic characteristics such as higher fecundity, faster maturation time, higher infectivity, increased pathology and the ability to infect both intermediate snail hosts of the parental species, thereby widening their intermediate host spectrum [19]–[20]. Whilst in nature host specificity and distribution may have restricted the potential for hybridization between schistosomes, natural and anthropogenic environmental changes, accompanied by host migration, introduction and/or radiation, can all serve to alter the distribution of schistosome species. This can result in novel interactions between schistosomes and host and also between different schistosome species infecting the same host. For example, dam construction and irrigation may be expected to give rise to changes in agricultural practice and create new habitats for intermediate snail hosts, bringing humans into even greater contact with their livestock, and hence the parasites of these animals, certain species of which can successfully hybridize with human schistosome species.

Due to the inaccessibility of schistosome adult worms within their hosts, detecting natural interactions and hybridization between schistosome species, especially in humans, can be highly problematic. Past studies have speculated on such events using morphological characteristics such as egg shape, infection site and snail compatibility, although this can be misleading [19]–[26]. Recent developments in the ability to store and genetically analyze individual schistosome larval stages directly from their natural hosts have, nevertheless, proved revolutionary in detecting natural hybridization events [27]–[33]. Accordingly, studies incorporating such molecular analysis of the parasites have proved that interactions and hybridization between schistosome species does occur in nature with varying epidemiological outcomes [19], [34]–[37]. In particular, a preliminary study reported on the bidirectional hybridization between a cattle schistosome, *S. bovis*, and a human schistosome, *S. haematobium*, in several villages along the Senegal River Basin in Northern Senegal [32]. Hybrid larval stages were found in human urine and stool samples and also transmitted through both the intermediate snail hosts (*Bulinus truncatus* and *B. globosus* respectively) of the parental species. This hybridization was hypothesized to have been able to occur due to the ecological and climatic changes that have taken place in these areas over the last 30 years facilitating the creation of areas of sympatry between these schistosome species and their hosts. Whilst this study provided the first conclusive evidence for the natural hybridization between these two species, it also inspired a number of further related questions such as: what is the occurrence, host use and distribution of these hybrids in Senegal and elsewhere. Additionally, in Senegal, there exists another schistosome species within the *S. haematobium* group, *S. curassoni*, which infects sheep, goats and cattle and is very closely related to both *S. bovis* and *S. haematobium*, potentially enabling all three species to interact if given the opportunity [38]–[41]. The definitive host range of *S. curassoni* and *S. bovis* does overlap, so it may be predicted that these two species also natural hybridize in areas of sympatry. Indeed an earlier report by Rollinson and colleagues [41], provided initial field and experimental evidence, based on isoenzyme data, for the hybridization between these two species in Senegal and Mali. There has also been some speculation about the potential involvement of *S. curassoni* infecting or interacting with *S. haematobium* in humans in Niger [42], although there was no conclusive evidence to support this as more sensitive molecular markers were needed to discriminate between *S. haematobium* and *S. curassoni* [41]–[44].

In the current study we use molecular sequencing tools and a multi-locus approach to provide novel data on the occurrence, interactions and host use of all three schistosome species and their natural hybrids at four foci across Senegal. In particular we aimed to confirm the presence of *S. curassoni* or hybrids thereof in children living in areas where *S. curassoni* occurs in ruminants and where transmission is likely to occur. We also aimed to elucidate the role of domestic livestock in the transmission and possible hybridization of the human schistosome *S. haematobium* and to provide data on the frequency and viability of the hybridization between, and also the transmission of, *S. haematobium*, *S. bovis* and *S. curassoni* at the specific field study sites. These data are discussed in relation to the implications for the transmission and control of schistosomiasis in Senegal and other neigbouring West African countries, where all three schistosome species may be occurring and interacting.

## Materials and Methods

### Parasite collection

**Figure 1:**
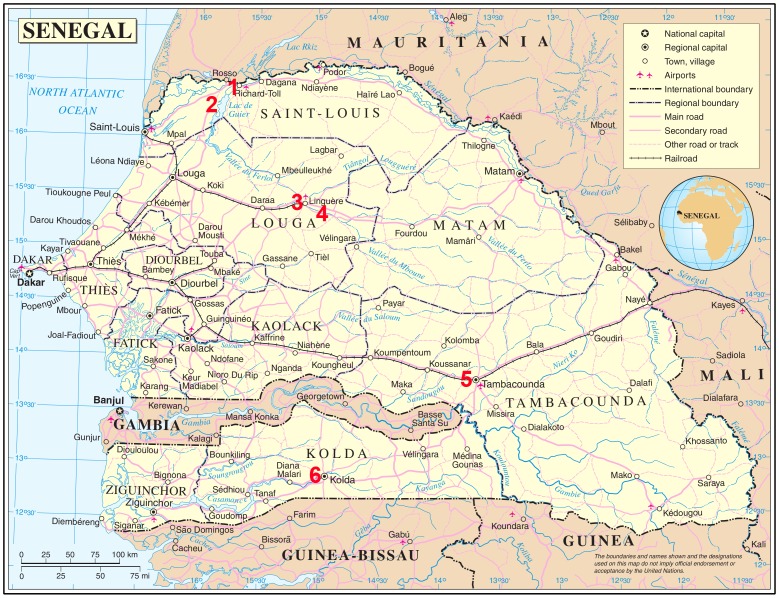
Map showing the location of the survey sites across Senegal. The survey sties are numbered in red. GPS coordinates: 1. Richard Toll = 16°27′40.71″N, 15°41′15.29″W, 2. Nder = 16°15′33.50″N, 15°53′2.13″W, 3. Linguiere = 15°23′34.33″N, 15°6′55.87″W, 4. Barkedji =  =  15°16′38.46″N, 14°51′55.48″W, 5. Tambacounda = 13°46′8.00″N, 13°40′2.00″W, 6. Kolda = 12°53′58.27″N, 14°56′39.37″W. The regions of Senegal are also shown on the map.

Parasitological surveys of domestic livestock and children were carried out in March 2009 and 2010 in four areas across Senegal; the Senegal River Basin (SRB) in the North, Vallée du Ferlo which is central, Tambacounda in the South East and Kolda in the South. (Figure 1). These sites were specifically selected as they were known foci for human and livestock schistosomiasis (personal communication). As these were mainly pilot surveys aimed to positively identify the different *Schistosoma* species and any hybrids infecting livestock and children in each area only human urine samples and animal intestines were sampled.

#### Collection of schistosome miracidia from children

To detect the presence and prevalence of urinary schistosomiasis, urine samples were collected from school-aged children in each area (refer to Table S1 for details). Schools and villages where children were suspected by the local health workers to have urinary schistosomiasis, were selected to take part in the study. Positive urine samples from each child were first identified using haemastix and by visual inspection to detect the presence of blood. Eggs from positive samples were hatched by sedimentation and subsequent exposure to clean water and light. Using a stereomicroscope individual miracidia were harvested and stored in RNA*later* as described by Webster [33].

#### Schistosome collection from domestic livestock

In each abattoir, intestines were randomly obtained from individual animals routinely slaughtered as part of the daily work of the abattoir. The mesenteric veins around the small and large intestines of individual cows, sheep or goats that had been slaughtered were visually inspected for the presence of schistosome worms. Any worms found were dissected from the veins and stored as individual pairs or individual single worms in ethanol. The number of worms dissected from each animal and the animal species was recorded. From some of the highly infected animals, a small piece of the liver was taken, macerated, and passed through a 212 µm sieve with 0.85% saline and any schistosome eggs present in the samples concentrated using a Pitchford funnel [45]. The eggs were then released into a clean Petri dish and exposed to fresh water and light to facilitate hatching. Individual miracidia were stored in RNA*later* as above.

### Genomic DNA (gDNA) extraction

gDNA from individual miracidia was extracted as described in Webster [33]. Individual worm pairs were washed in TE buffer to remove any residual ethanol and allowed time to relax in the TE buffer so that they could be separated. The male and female worm from each pair was recorded. gDNA from individual worms was extracted using the DNeasy blood and tissue kit (Qiagen) according the manufacture's protocol and DNA was eluted in a total of 100 µl.

To enable high throughput processing of the samples the majority of the gDNA extractions were carried out using the DNeasy blood and tissue kit 96 well plate spin protocol (Qiagen) according the manufacturer's protocol and DNA was eluted in a total of 100 µl.

### Molecular hybrid detection

Hybrid detection of schistosomes requires a multi-locus approach, analyzing both mitochondrial and nuclear DNA simultaneously from individual specimens [32]. A partial region of the mitochondrial *cox*1 gene and the complete ITS1+2 rDNA were analyzed from each individual as described below.

#### Mitochondrial (mt) cox1 amplification and analysis

To identify individual miracidia and worms with *S. haematobium*, *S. bovis* or *S. curassoni* mtDNA, the *cox*1 rapid diagnostic multiplex PCR (RD-PCR) was carried out for each individual sample as described by Webster and colleagues [46]. 4 µ1 of each RD-PCR was visualised on a 2% gel-red agarose gel. Any miracidium that was collected from the human urine samples that did not present a clear *S. haematobium* RD-PCR profile was selected for sequencing and those that did present a clear *S. haematobium* profile were recorded as having *S. haematobium* mtDNA. In addition, a selection of these miracidia (4 from each urine sample) was also sequenced to confirm the mtDNA identity. Where there was a mixture of *S. bovis* and *S. curassoni* RD-PCR profiles from the individual worms all RD-PCR products were sequenced to confirm the mtDNA identity. All RD-PCR's were run in conjunction with +ve and −ve reactions.

#### Nuclear ITS1+2 rDNA amplification

The complete ITS1+2 rDNA (981 bp) was amplified from each individual specimen in 25 µ1reactions containing 2 µ1 of each gDNA extract, Illustra PuReTaq Ready-To-Go PCR Beads (GE Healthcare) and 10 pmol of each forward and reverse primer [32]. Thermal cycling was performed in a Perkin Elmer 9600 Thermal Cycler and the PCR conditions used were: 5 min denaturing at 95°C: 40 cycles of 30 sec at 95°C, 30 sec at 40°C, 1 min at 72°C; followed by a final extension period of 7 min at 72°C. 4 µl of each PCR reaction was visualised on a 0.8% gel-red agarose gel to confirm amplification success. All PCR's were run in conjunction with +ve and −ve reactions.

#### Sequencing and analysis

Selected RD-PCR's and all positive ITS PCR reactions were purified using the Qiaquick 96 PCR purification Kit (Qiagen) according to the manufacturer's protocol. Both the forward and reverse strands of the purified amplicons were sequenced using a dilution of the original PCR primers and a Fluorescent Dye Terminator Sequencing Kit (Applied Biosystems), the sequencing reactions were run on an Applied Biosystems 377 automated sequencer. For the RD-PCR amplicons, the primer used for the reverse sequencing reaction corresponded with the diagnostic profile of the amplicon.

The sequences were assembled and manually edited using Sequencher V.4.5 (GeneCodes Corp.). Identity of the sequence was confirmed using the Basic Local Alignment Search Tool (BLAST) and comparison to the reference sequences (see below). At the polymorphic positions of the ITS1+2 between *S. haematobium*, *S. bovis* and *S. curassoni*, any occurrence of double chromatogram peaks was recorded to identify mixed ITS sequences. The species identity of the mt and nuclear DNA for each individual specimen was recorded (Tables S1+S2).

### Reference sequences

The *cox1* and ITS reference sequences of *S. haematobium*, *S. bovis* and *S. curassoni* from Huyse and colleagues [32], were used to identify and compare the sequences from the samples in this study.

### Crosses of *S. haematobium*, *S. bovis* and *S. curassoni* in laboratory animals

During the collection of the parasite material at the sampling sites, eggs were also collected to establish laboratory isolates of *S. haematobium*, *S. bovis* and *S. curassoni.*

#### Group 1: *S. curassoni*

A small liver sample was taken from a cow (C1, Table S2) from Tambacounda and processed for miracidial collection. After an hour of hatching a few miracidia had hatched and were seen swimming around the Petri dish. In view of the low numbers and slowness of the hatching, eight laboratory bred *Bulinus wrighti* were added to the Petri dish and left for approximately 12 hours to facilitate snail infection.

#### Group 2: *S. bovis*

A small liver sample was taken from a cow (C4, Table S2) from Kolda and processed for miracidial hatching. The eggs hatched well and 12 laboratory bred *Bulinus wrighti* were exposed to five miracidia each.

#### Group 3: *S. haematobium*

Miracidia hatched from the urine sample collected from a child ID (32, Table S1) from Nder school in the Senegal River basin were used to infect 24 laboratory bred *Bulinus wrighti.* The snails were individually exposed to five miracidia each.

#### Snail shedding and molecular species identification

The snails were maintained in their groups in the NHM schistosome culture facility and at 4–6 weeks post exposure each group was exposed to light to facilitate cercarial shedding. At the time of the snail infections, the species identity of the miracidia used had not been confirmed so 20 individual cercariae from each group of snails were harvested for molecular identification. Individual cercariae were captured in 2–3 µl of water and pipetted into individual Eppendorf tubes. gDNA was immediately extracted from each individual cercaria using the DNeasy blood and tissue kit (Qiagen) according to the manufacturer's protocol, but with the modifications to the protocol as described in Webster [33]. The mt *cox*1 and nuclear ITS DNA were analysed and identified from each individual cercaria using the same methodology as described earlier.

#### Species crosses in laboratory animals

The remaining cercariae from each group of snails were used to infect two laboratory mice or two laboratory hamsters (see Table 1) by the paddling technique [17]. The snails were then set up to shed cercariae again every other day and the same animals were exposed to the cercariae from a different group of snails, so that each of the animals had been exposed to a combination of cercariae from two of the different groups of snails (see Table 1). If necessary, animals were repeatedly exposed to cercariae shed from the snails on different days so that they had been exposed to approximately 100 cercariae from each of the 2 snail groups to which they had been initially exposed. Ten weeks post exposure, animals were culled and all the worms were perfused and dissected from the animals. Each individual worm pair was put into a separate pot in 0.85% saline and allowed to separate. gDNA was directly extracted from each individual worm and the mt *cox*1 and nuclear ITS DNA were analysed and identified as described earlier. The molecular identity of each male and female worm in each pair was recorded. From each animal the liver was processed for miracidial hatching [17] and 96 miracidia were harvested individually from each animal cross for molecular identification. Each miracidia was captured individually in 2–3 µl of water and pipetted into a single well of a 96 well PCR plate. gDNA was extracted from each individual miracidium as described in Webster [33]. The mt *cox*1 and nuclear ITS DNA were analysed and identified from each individual miracidium using the same methodology as previously described.

**Table 1:** Experimental laboratory animal infections.

Animal cross (No. of animals)	1^st^ infection		2^nd^ infection		Homospecific worm pairs (no.)	No. of homospecific miracidia (no.)	Heterospecific worm pairs (no)	Hybrid miracidia**		
	Snail Group	Cercariae sp.	Snail Group	Cercariae sp.				ITS profile	mt *cox*1 profile	No.
1 (2 Mice)	1	*S. c*	2	*S. b*	*S. b* M X *S. b* F (14)	18	*S. b* M X *S. c* F (15)	*S. c*/*S. b*	*S. c*	15
					*S. c* M X *S. c* F (41)	51	*S. c* M X *S. b* F (17)	*S. b/S. c*	*S. b*	12
2 (2 Hamsters*)	2	*S. b*	3	*S. h*	*S. b* M X *S. b* F (11)	83	*S. h* M X *S. b* F (3)	*S. h*/*S. b*	*S. b*	5
					*S. h* M X *S. h* F (4)	4	*S. b* M X *S. h* F (2)	*S. b/S. h*	*S. h*	4
3 (2 Hamsters*)	3	*S. h*	1	S. c	*S. c* M X *S. c* F (56)	77	*S. h* M X *S. c* F (7)	*S. h*/*S. c*	*S. c*	3
					*S. h* M X *S. h* F (24)	12	*S. c* M X *S. h* F (8)	*S. c/S. h*	*S. h*	4

* Hamsters were used as *S. haematobium* does not develop well in mice.

** Genetic profiles and number of hybrid miracidia resulting from the heterospecific crosses. In total 96 miracidia from each animal cross were genetically analysed.

*S. c* = *S. curassoni*, *S. b* = *S. bovis*, *S. h* = *S. haematobium*, M = male and F = female.

### Ethics statement

Ethical approval for these studies was obtained from the Imperial College Research Ethics Committee (ICREC), Imperial College London and the Ministry of Health Dakar, Senegal. Before conducting the study, the MoH-approved plan of action had been presented and adopted by regional and local administrative and health authorities. Meetings were held in each village to inform the village leader, heads of the families, local health authority, teachers, parents and children about the study, its purpose and to invite them to participate voluntarily. According to common practice and with approval from the Imperial College Research Ethics Committee (ICREC), due to low levels of literacy all village leaders, teachers, parents and study participants gave oral consent for the studies to take place. Informed consent for the urine examinations was obtained from each study participant and their parents or guardians. Oral consent for each participant was documented by inscription at school committees comprising of parents, teachers and community leaders. All the data were analysed anonymously and all schistosomiasis positive participants were treated with PZQ (40 mg/kg). In schools or classes where the percentages of infections were more than 50%, mass treatment of all children was carried out at the end of the study.

Laboratory animal use was within a designated facility regulated under the terms of the UK Animals (Scientific Procedures) Act, 1986, complying with all requirements therein, including an internal ethical review process at the NHM and regular independent Home Office inspection. Work was carried out under the Home Office project license number 70/6834.

## Results

Adult schistosome worms and miracidia collected during parasitological surveys across Senegal of domestic livestock and children at four transmission foci, identified as potential hybrid zones, were identified by nuclear and mitochondrial (mt) DNA genotyping. The numbers of *S. haematobium*, *S. bovis*, *S. curassoni* or hybrids thereof from each host were recorded. The viability of the interactions between *S. haematobium*, *S. bovis* and *S. curassoni* were also investigated during inter-species crossing experiments in laboratory rodents.

### Nuclear and mtDNA genotyping

#### ITS1+2 nuclear rDNA

In the reference sequences and all the specimens examined *S. haematobium* differed from *S. bovis* and *S. curassoni* at five point mutations and *S. curassoni* differed from *S. bovis* at one point mutation. No intra-specific variation was detected in all the ITS sequences, however, in some of the hybrid miracidia double chromatogram profiles were observed at the polymorphic positions between the two species involved in the hybridization. The peak height that represented each species varied, with some hybrids having a higher peak for one species than the other and some hybrids having equal peak heights for both species.

#### *cox*1 mtDNA

Considerable genetic distance separates the *cox*1 mtDNA of *S. haematobium*, *S. bovis* and *S. curassoni* and this is therefore a good molecular tool for mtDNA identification for these three species [32]. The diagnostic *cox*1 RD-PCR [46] proved robust for distinguishing worms and larval stages with *S. haematobium* mtDNA from those that had *S. bovis* and *S. curassoni* mtDNA, with all the individuals that produced a *S. haematobium cox*1 RD-PCR profile, that were sequenced, having a *S. haematobium cox*1 mtDNA sequence. The diagnostic *cox*1 RD-PCR varied in its robustness to distinguish between worms and larval stages with *S. bovis* and *S. curassoni* mtDNA and hence all the individuals that presented a *S. bovis* or a double-banded (both the *S. haematobium* and *S. bovis* diagnostic bands present [46]) *cox*1 RD-PCR profile were sequenced. All individuals that presented a double-banded *cox*1 RD-PCR profile had a *cox*1 mtDNA sequence that was identified as *S. curassoni.* The majority of individuals that presented a *S. bovis* RD-PCR *cox*1 profile had a *cox*1 mtDNA sequence that was identified as *S. bovis*, however, a few had a *cox*1 mtDNA sequence that was identified as *S. curassoni*.

### Human parasitological surveys

The prevalence of human urogenital schistosomiasis was high in all areas sampled, ranging from 57–100%, and visible haematuria was obvious in urines samples from all study areas. A total of 823 individual miracidia were collected and genetically analysed from 52 urine samples. 79% of the miracidia were molecularly identified as pure *S. haematobium*, however, 21% presented a mixed mt *cox*1 + nuclear ITS genotype suggesting that these miracidia had a hybrid origin. Hybrid miracidia were found in 88% of the urine samples analysed and in all areas surveyed. The numbers and type of hybrids varied between urine samples and areas (Table S1). Hybrids between *S. bovis* and *S. haematobium* were found in children from all areas except Barkedji in the Vallée du Ferlo and hybrids between *S. curassoni* and *S. haematobium* were only isolated in Tambacounda and the Vallée du Ferlo.

### Domestic livestock parasitological surveys

In total, 1004 schistosome worms (502 pairs) were dissected and genetically analysed from the mesenteric vessels of 33 animal intestines and 109 miracidia were hatched and analysed from three infected liver samples (Table S2). Only *S. bovis* was found in animals from Kolda and Richard Toll but *S. bovis* and *S. curassoni* were found in animals from Tambacounda and the Vallée du Ferlo. The number of worm pairs found in each individual animal varied considerably from 1 to over 100. *S. bovis*/*S. curassoni* hybrid worms with different genetic profiles were found in small numbers in the intestines of five animals from Tambacounda and all these hybrids were paired with either *S. bovis* or *S. curassoni* worms. No worms with pure or hybrid *S. haematobium* genetic profiles were found in any domestic animal.

### Inter-species crosses in laboratory rodents

All three species were successfully isolated from the field samples and maintained in laboratory *Bulinus wrighti* snails. The molecular ITS and *cox*1 identification of the cercariae resulting from the three groups of laboratory snail infections showed that each group was infected with one species (Group 1 *S. curassoni*, Group 2 *S. bovis* and Group 3 *S. haematobium*). No hybrid profiles were observed in these isolates. The mixed species infections in all the laboratory rodents (animal crosses 1–3, Table 1) produced heterospecific pairs between male and female worms of all three species (Table 1) and also homospecific pairs. The numbers of pairs varied between animals with the most abundant heterospecific parings occurring between *S. bovis* and *S. curassoni* worms. Miracidia were hatched from the infected livers of animals with mixed infections. Molecular typing of these miracidia detected hybrid offspring (Table 1) and confirmed viable heterospecific pairings between all three species. Homospecific miracidia of each species were also identified. The numbers of each type of miracidia correlated to the number of homospecific or heterospecific worm pairs present.

## Discussion

When humans and their livestock start to frequent the same water bodies, due to environmental and/or behavioural changes, novel zoonotic hybrid schistosomes could evolve with subsequent changes in the parasite's life history traits, transmission potential and virulence. Here, through incorporating both field and laboratory experimental data, we provide both additional and novel molecular evidence for the natural hybridization between three *Schistosoma* species in Senegal: *S. haematobium* a parasite of humans, *S. bovis* and *S. curassoni* which both parasitize primarily domestic livestock. The data from different areas of Senegal demonstrate that these hybridisation events are not rare and so, understanding such inter-species interactions will be essential for predicting the outcomes of current and future control programmes in hybrid zones.

### Hybridization and introgression

Children were found excreting *S. haematobium*/*S. curassoni* hybrids in Tambacounda and the Vallée du Ferlo and *S. haematobium*/*S. bovis* hybrids in Tambacounda, Kolda and the SRB. A low number of *S. bovis* and *S. curassoni* hybrid worms were found in cows slaughtered in the Tambacounda abattoir but no *S. haematobium* worms or hybrids thereof were found in ruminants, however this could be due to the fact that only the intestines of the animals were sampled. . These data do suggest that at some point host switching has been able to take place between these three sister species enabling the two species involved to interact, hybridize and produce viable offspring.

The laboratory mixed infection experiments further indicate that males and females of each species readily pair and produce viable hybrid offspring and in these experimental crosses there does not appear to be any competition, exclusion or mating preference between the species and each cross produced viable hybrid offspring.

In each of the three types of hybrids two hybrid lines were observed, resulting from bidirectional introgressive hybridization with mtDNA from both the parental species introgressing into the other species involved in the hybridization (see Table S1+S2). Regarding the nuclear DNA profiles, initial hybrid generations usually display both parental nuclear rDNA ITS copies, resulting in double chromatogram peaks at the species-specific mutation sites [32], [47]–[49]. In subsequent hybrid generations or backcrossing of hybrids with parental species, biased homogenisation towards one of the parental species may result in nuclear rDNA ITS sequences that can appear as just one species or the other [49]. As the dynamics of this homogenisation and silencing of the genetic signal from one species over the other is unknown it is impossible to decipher which generation the natural hybrids are from by looking at their genetic profiles. Nevertheless, the observation of both pure and mixed nuclear rDNA ITS sequences within our hybrid populations strongly suggest that there are different generations of hybrids and/or hybrid backcrosses persisting in nature.

### Host switching in the definitive hosts and animal reservoirs of infection

The hybridization of *S. bovis* and *S. curassoni* with *S. haematobium* is of particular interest, as for this to occur, host switching must have taken place of either, *S. bovis* and *S. curassoni* into humans or *S. haematobium* into domestic livestock. The oviposition site of a schistosome pair is generally assumed to primarily be dependent on the species of the male worm [37], [43], with *S. bovis* and *S. curassoni* males carrying their females to the intestinal tract and *S. haematobium* males carrying their females to the urinary tract. It is unknown how hybridization will affect this phenotypic/behavioural characteristic, however it would be expected that in the initial parental cross the worm pair would migrate to the oviposition site determined by the species of the male worm. Hybrid miracidia analysed from the human urine samples that present *S. curassoni cox*1 and mixed ITS sequences or *S. bovis cox*1 and mixed ITS sequences could be first generation hybrids resulting from pure parental crosses between *S. haematobium* males and *S. curassoni* or *S. bovis* females, however, it is also possible that second generation hybrids or hybrid backcrosses could also present these genetic profiles. It would be expected that first generation hybrids of the reciprocal cross (*S. haematobium* females paired with males of *S. bovis* or *S. curassoni*) would be excreted in stool samples, therefore miracidia from any eggs present in stool samples also need to be analysed to identify further hybrids and also any possible homospecific infections of *S. bovis* and or *S. curassoni* in humans.

No *S. haematobium*/*S. bovis* or *S. haematobium*/*S. curassoni* hybrids or *S. haematobium* worms were found in the domestic livestock, possibly suggesting that *S. haematobium* and indeed *S. haematobium* hybrids may lack the ability to penetrate and or develop in ruminants. It is also necessary to consider whether major differences in the vasculature between ruminants and humans would restrict the migration of *S. haematobium* to the blood vessels of the bladder of ruminants. However, as only the intestinal tracts of the slaughtered animals were routinely available for inspection at the abattoirs, and as *S. haematobium* is a parasite of the urinary tract, the presence of *S. haematobium* or the hybrids may have been missed. A much earlier study in Zambia did report the possible finding of *S. haematobium*/*S. mattheei* (another closely related ruminant schistosome) hybrids in the mesenteric veins of cattle [50] however these findings remain unconfirmed. It is clear that more extensive and detailed dissections off and egg collections from the ruminants is warranted.

The infectivity of a schistosome to a mammalian host depends on several physical and immunological factors, however, the variety of host use by different species of the *Schistosoma* genus suggests that host switching has occurred at several time points in the evolution on this genus [38]. It is possible that the close phylogenetic ancestral position of *S. haematobium* to its sister species *S. curassoni* and *S. bovis*, together with physical and anatomical host differences may enable the latter two species to retain an ability to infect humans. Another possibility is that the initial pairing between these human and domestic livestock parasites occurred first in another susceptible host, such as a rodent. Rodents have proved extremely efficient for passaging a variety of schistosome species in the laboratory [17] and are utilised as reservoir hosts by other species of schistosome [51]–[54].

The hybridization between *S. curassoni*/*S. bovis* is not that surprising given their neighbouring phylogenetic position and their overlap in definitive host associations [38] and the molecular data presented here conclusively confirm that these parasites are able to hybridize.

### Distribution and prevalence

Observations from the different survey sites clearly demonstrate the focality of the transmission of the different species and their hybrids. Hybridization between the human and domestic livestock schistosomes appears common with hybrid genetic profiles recovered from human urine samples from several areas where the different species are transmitted sympatrically. Also, *S. haematobium*/*S. bovis* hybrids have previously been sampled from humans from several widely dispersed villages along the SRB [32], and the possibility of the hybridization between *S. haematobium/S. bovis* or *S. curassoni* was reported by [42] in Niger. Due to the wide distribution of the intermediate snail hosts *B. globosus* and *B. truncatus*, *S. haematobium* and *S. bovis* are common infections across much of Senegal, providing the opportunity for these species to interact, however the distribution of *S. curassoni*, transmitted through *B. umbilicatus*, is more restricted. The data presented here confirm an intense *S. curassoni* focus in the Vallée du Ferlo, increasing the known distribution of this species and the hybrids thereof. *S. haematobium/S. curassoni* hybrids were found in the urines of children sampled from the village of Barkedji, where only *S. curassoni* was found in the slaughtered sheep. This, together with the data from Tambacounda, provides the first confirmation of this species or hybrids thereof infecting children in Senegal.

Only small numbers of *S. curassoni/S. bovis* hybrids were found in cattle at Tambacounda abattoir, with most worms being identified as *S. curassoni* in agreement with an earlier report [41]. There are no obvious isolating barriers other than intermediate snail host preferences that prevent *S. curassoni* and *S. bovis* from interacting and hybridizing as both species readily infect ruminants. However, transmission of these two species does appear to be localised, with the majority of the animals sampled in one particular place being infected with either *S. bovis* or *S. curassoni.* The origins of the animals sampled at the small abattoirs in this study are usually unknown and would be almost impossible to trace due to changes of ownership during an animal's lifetime. The worm burden reflects past exposure: *S. bovis* transmission being associated with *B. truncatus* habitats while *S. curassoni* is associated with *B. umbilicatus* [54].

With regard to the role of intermediate snail hosts, hybridization between *Schistosoma* species potentially enables the schistosomes to increase their host range with the hybrids being able to utilize both the intermediate snail hosts of the parental species, which will have important implications for schistosomiasis epidemiology by increasing transmission and distribution [19]–[20], [27], [32]. Snail surveys were not conducted during this study, however, the study of Huyse and colleagues [32] did provide evidence for the transmission of *S. haematobium/S. bovis* hybrids through both *B. globosus* and *B. truncatus* the intermediate snail hosts of *S. haematobium* and *S. bovis* respectively. Snail surveys and molecular screening of cercariae from the hybrid zones are needed to clarify what role each snail species plays in the transmission of the parental species and their hybrids.

Some degree of sympatric transmission enabling interbreeding between these species may have always occurred but remained undetected due to the limitations of previously available sampling and analysis tools. However, hybridisation between species can be further facilitated by the loss of ecological barriers existing between species due to natural and or man-made changes. [35]–[37]. In Northern Senegal, it was speculated that the hybridization between *S. haematobium* and *S. bovis* was facilitated by the creation of water bodies for agriculture, through dam construction, which led to an increased prevalence and distribution of the intermediate snail hosts and movements of humans and livestock to these resources, creating sympatric transmission of these species, resulting in hybridization. In the additional hybrid zones confirmed in this study, while no such ecological change can be attributed to enabling hybridization to have taken place, the natural progression in farming, population (both human and livestock) movements and expansion will result in areas of increased close associations between humans and their domestic livestock, increasing the chances of interspecific interactions between the schistosomes they carry. Other behavioural factors could also have an important impact, for instance during the sampling of cattle in the abattoir in Richard Toll in the SRB where *S. bovis* was highly prevalent, it became apparent that due to the lack of running water the intestines of slaughtered animals were routinely washed in the local river, which was also frequented by the local people for their everyday activities, thus creating a potential sympatric transmission site for *S. haematobium* and *S. bovis*.

### Implications

Introgressive hybridization may lead to phenotypic changes that can dramatically influence disease dynamics and evolution of the parasites. Although treatment with praziquantel, the drug routinely used to control human schistosomiasis across Africa, is successful against *S. haematobium*, *S. bovis* and *S. curassoni* [55]–[56], hybridization between different *Schistosoma* species have been reported to affect the success of drug treatment in cattle [57], cause severe disease outbreaks and competitive exclusion of one species by the other [35]–[37] and laboratory hybrids have been observed to acquire enhanced characteristics such as infectivity, fecundity and growth rates [19], [20], [54].

*S. haematobium* has long been recognised as a parasite with few, if any, reservoir hosts [38]. However, it seems that in Senegal and possibly in other areas of West Africa, new genotypes may emerge that may pass from people to domestic livestock and *vice versa*. Furthermore, if the hybridization events reported here result in phenotypic characteristics that influence drug sensitivity, pathology and transmission, it will be highly important to re-evaluate control strategies in these hybrid zones. The increased host range of the hybrid parasites and changes in host distribution may have a direct impact on transmission of these schistosomes. Human and veterinary schistosomiasis in Senegal and neighbouring countries needs to be further monitored to clarify further the epidemiology and dynamic interactions of these closely related schistosome species.

## Supporting Information

Table S1 Data from children.
(DOC)

Table S2 Date from domestic livestock.
(DOC)
