# Supplementary material for: Introgressive Hybridization of Schistosoma haematobium Group Species in Senegal: Species Barrier Break Down between Ruminant and Human Schistosomes
Source: PLoS Negl Trop Dis. 2013 Apr 4;7(4):e2110. doi: 10.1371/journal.pntd.0002110 (PMC3617179; doi:10.1371/journal.pntd.0002110)
Supplement: Table S1 — Data from children. (DOC) [file pntd.0002110.s001.doc]

|  |  |  |  |  | **No. of miracidia that presented the different genetic profiles** | | | | | | | | | | |
| --- | --- | --- | --- | --- | --- | --- | --- | --- | --- | --- | --- | --- | --- | --- | --- |
|  |  |  |  |  | **Pure species** | | | **Hybrids** | | | | | | | |
|  | | | | | ***S.h*** | ***S.c*** | ***S.b*** | ***S.h* / *S.c*** | | | | ***S.h* / *S.b*** | | | |
| **Area** | **Village/ School** | **No. urines** | **% infection** | **Child ID** | ***S.h*(*cox*1)**  ***S.h*(ITS)** | ***S.c*(*cox*1)**  ***S.c*(ITS)** | ***S.b*(*cox*1)**  ***S.b*(ITS)** | ***S.c*(*cox*1)**  ***S.h*(ITS)** | ***S.h*(*cox*1)**  ***S.c*(ITS)** | ***S.c*(*cox*1)**  **Mix(ITS)** | ***S.h*(*cox*1)**  **Mix(ITS)** | ***S.b*(*cox*1)**  ***S.h*(ITS)** | ***S.h*(*cox*1)**  ***S.b*(ITS)** | ***S.b*(*cox*1)**  **Mix(ITS)** | ***S.h*(*cox*1)**  **Mix(ITS)** |
| Tambacounda | Majed Dibi | 24 | 96 | MD17 | 8 |  |  | 1 |  |  | 2 |  |  |  | 1 |
|  |  |  |  | MD16 | 10 |  |  |  |  |  |  |  |  |  |  |
|  |  |  |  | MD19 | 10 |  |  |  |  |  |  |  |  |  |  |
|  |  |  |  | MD20 | 9 |  |  |  | 1 | 1 | 2 |  |  | 1 |  |
|  | Sinthiou Malem | 142 | 87 | SM20 | 4 |  |  | 2 |  |  | 1 | 1 |  |  | 2 |
|  |  |  |  | SM46 | 5 |  |  | 2 | 1 |  |  |  | 2 |  | 1 |
|  |  |  |  | SM67 | 7 |  |  |  |  |  |  | 1 |  |  |  |
|  |  |  |  | SM128 | 8 |  |  |  |  |  | 1 |  | 1 |  |  |
|  | Thiawor | 55 | 80 | T26 | 7 |  |  |  |  |  | 2 |  |  |  | 1 |
|  |  |  |  | T28 | 7 |  |  |  | 1 |  | 1 |  |  |  | 1 |
|  |  |  |  | T32 | 8 |  |  | 1 |  |  |  | 1 |  |  |  |
|  |  |  |  | T65 | 8 |  |  |  |  | 2 |  |  |  |  |  |
| Kolda | Sara Sara | 14 | 57 | SS1 | 4 |  |  |  |  |  |  |  | 6 |  |  |
|  |  |  |  | SS2 | 8 |  |  |  |  |  |  |  |  | 1 | 3 |
|  |  |  |  | SS10 | 17 |  |  |  |  |  |  |  |  | 1 |  |
| Middle Valley of the Senegal River Basin | Nder | 83 | 64 | N74 | 11 |  |  |  |  |  |  |  |  |  | 1 |
|  |  |  |  | N85 | 12 |  |  |  |  |  |  |  |  |  | 2 |
|  |  |  |  | N65 | 12 |  |  |  |  |  |  |  |  |  | 1 |
|  |  |  |  | N55 | 14 |  |  |  |  |  |  |  |  |  |  |
|  |  |  |  | N56 | 11 |  |  |  |  |  |  |  |  |  |  |
|  |  |  |  | N1 | 9 |  |  |  |  |  |  | 2 |  |  | 1 |
|  |  |  |  | N11 | 10 |  |  |  |  |  |  |  | 1 |  | 1 |
|  |  |  |  | N16 | 10 |  |  |  |  |  |  |  | 2 |  |  |
|  |  |  |  | N19 | 21 |  |  |  |  |  |  |  |  |  | 3 |
|  |  |  |  | N20 | 12 |  |  |  |  |  |  |  | 2 |  |  |
|  |  |  |  | N23 | 22 |  |  |  |  |  |  |  |  |  | 2 |
|  |  |  |  | N27 | 15 |  |  |  |  |  |  | 1 |  |  | 1 |
|  |  |  |  | N30 | 10 |  |  |  |  |  |  |  |  | 2 |  |
|  |  |  |  | N32 | 20 |  |  |  |  |  |  |  |  |  |  |
|  |  |  |  | N37 | 5 |  |  |  |  |  |  |  | 4 |  | 3 |
|  |  |  |  | N38 | 7 |  |  |  |  |  |  | 3 |  | 4 |  |
|  |  |  |  | N47 | 9 |  |  |  |  |  |  |  |  |  | 1 |
|  |  |  |  | N49 | 30 |  |  |  |  |  |  |  | 1 | 1 |  |
|  |  |  |  | N52 | 6 |  |  |  |  |  |  |  |  |  |  |
|  |  |  |  | N53 | 10 |  |  |  |  |  |  | 2 |  | 1 | 2 |
|  |  |  |  | N55 | 21 |  |  |  |  |  |  |  |  |  | 3 |
|  |  |  |  | N56 | 20 |  |  |  |  |  |  | 1 |  | 1 |  |
|  |  |  |  | N59 | 9 |  |  |  |  |  |  |  | 2 |  | 4 |
|  |  |  |  | N60 | 10 |  |  |  |  |  |  | 2 |  |  | 1 |
|  |  |  |  | N85 | 11 |  |  |  |  |  |  |  |  |  | 1 |
| Vallée du Ferlo | Barkedji | 17 | 100 | B11 | 24 |  |  |  | 2 |  | 1 |  |  |  |  |
|  |  |  |  | B35 | 22 |  |  |  | 1 | 1 | 2 |  |  |  |  |
|  |  |  |  | B47 | 21 |  |  |  | 1 |  |  |  |  |  |  |
|  |  |  |  | B22 | 23 |  |  | 1 | 3 |  | 1 |  |  |  |  |
|  |  |  |  | B45 | 17 |  |  |  | 1 | 1 |  |  |  |  |  |
|  |  |  |  | B24 | 22 |  |  |  | 1 |  | 2 |  |  |  |  |
|  |  |  |  | B60 | 32 |  |  | 1 |  |  | 3 |  |  |  |  |
|  |  |  |  | B42 | 24 |  |  |  | 3 | 1 |  |  |  |  |  |
|  |  |  |  | B49 | 10 |  |  |  | 3 |  |  |  |  |  |  |
|  |  |  |  | B33 | 21 |  |  |  | 1 |  | 2 |  |  |  |  |
|  |  |  |  | B37 | 12 |  |  |  | 3 | 2 |  |  |  |  |  |
|  |  |  |  | B27 | 6 |  |  | 1 |  |  |  |  |  |  |  |
| Totals | | | | 52 | 681 | 0 | 0 | 9 | 22 | 8 | 20 | 14 | 21 | 12 | 36 |

*S.h* = *S. haematobium, S.c* = *S. curassoni, S.b = S. bovis*
