# Supplementary material for: Introgressive Hybridization of Schistosoma haematobium Group Species in Senegal: Species Barrier Break Down between Ruminant and Human Schistosomes
Source: PLoS Negl Trop Dis. 2013 Apr 4;7(4):e2110. doi: 10.1371/journal.pntd.0002110 (PMC3617179; doi:10.1371/journal.pntd.0002110)
Supplement: Table S2 — Date from domestic livestock. (DOC) [file pntd.0002110.s002.doc]

|  | | | | **No. of worms and miracidia that presented the different genetic profiles** | | | | | |
| --- | --- | --- | --- | --- | --- | --- | --- | --- | --- |
|  | | | | **Pure species** | | **Hybrids** | | | |
|  | | | | ***S.b*** | ***S.c*** | ***S.c* / *S.b*** | | | |
| **Area** | **Village or Abbatoir** | **Animal*** | **No. worm pairs (P) or miracidia (m)** | ***S.b*(*cox*1) *S.b*(ITS)** | ***S.c*(*cox*1) *S.c*(ITS)** | ***S.c*(*cox*1) *S.b*(ITS)** | ***S.b*(*cox*1)**  ***S.c*(ITS)** | ***S.c*(*cox*1)**  **Mix(ITS)** | ***S.b*(*cox*1)**  **Mix(ITS)** |
| Middle Valley of the Senegal River Basin | Richard Toll | RTC1 | 3P | 6 |  |  |  |  |  |
|  |  | RTC2 | 3P | 6 |  |  |  |  |  |
|  |  | RTC3 | 26P | 52 |  |  |  |  |  |
|  |  | RTC4 | 3P | 6 |  |  |  |  |  |
|  |  | RTC5 | 11P | 22 |  |  |  |  |  |
|  |  | RTC6 | 8P | 16 |  |  |  |  |  |
|  |  | RTC7 | 1P | 2 |  |  |  |  |  |
|  |  | RTC8 | 8P | 16 |  |  |  |  |  |
|  |  | RTC9 | 8P | 16 |  |  |  |  |  |
|  |  | RTC10 | 8P | 16 |  |  |  |  |  |
|  |  | RTC11 | 8P | 16 |  |  |  |  |  |
| Kolda | Kolda | KC1 | 19P | 38 |  |  |  |  |  |
|  |  | KC2 | 3P + 3m | 9 |  |  |  |  |  |
|  |  | KC4 | 10m | 10 |  |  |  |  |  |
|  |  | KC4 | 24P | 48 |  |  |  |  |  |
|  |  | KC5 | 10P | 20 |  |  |  |  |  |
|  |  | KC6 | 2P | 4 |  |  |  |  |  |
| Tambacounda | Tambacounda | TS1 | 2P |  | 4 |  |  |  |  |
|  |  | TS2 | 6P |  | 12 |  |  |  |  |
|  |  | TC1 | 8P |  | 16 |  |  |  |  |
|  |  | TC2 | 58P |  | 113 | 3 |  |  |  |
|  |  | TC3 | 14P |  | 26 | 2 |  |  |  |
|  |  | TC4 | 18P |  | 32 |  |  | 5 |  |
|  |  | TC5 | 2P |  | 4 |  |  |  |  |
|  |  | TC6 | 2P | 3 |  |  |  |  | 1 |
|  |  | TC7 | 27P |  | 54 |  |  |  |  |
| Vallée du Ferlo | Barkedji | BS1 | 96P + 96m |  | 288 |  |  |  |  |
|  |  | BS2 | 16P |  | 32 |  |  |  |  |
|  | Linguiere | LG1 | 16P |  | 31 |  |  |  |  |
|  |  | LC1 | 32P | 64 |  |  |  |  |  |
|  |  | LG2 | 1P |  | 2 |  |  |  |  |
|  |  | LC2 | 4P | 8 |  |  |  |  |  |
|  |  | LS1 | 55P |  | 110 |  |  |  |  |
| Total | | 33 | 454P + 109m | 378 | 628 | 5 | 0 | 5 | 1 |

*C = Cow, S = Sheep, G= Goat

*S.c* = *S. curassoni, S.b = S. bovis*
